# Incidence, molecular characterization and phylogenetic diversity of phytoplasmas associated with brinjal little leaf disease in Maharashtra and Karnataka, India

**DOI:** 10.3389/fpls.2026.1870998

**Published:** 2026-06-29

**Authors:** Sarika Bhalerao, Vaishnavi Tattapure, Mahendra Rai, Nanjundappa Manjunatha, Sylwia Okoń

**Affiliations:** 1 Department of Plant Biotechnology, Vilasrao Deshmukh College of Agricultural Biotechnology, Latur, Vasantrao Naik Marathwada Krishi Vidyapeeth, Parbhani, Maharashtra, India; 2 Department of Microbiology, Nicolaus Copernicus University, Torun, Poland; 3 Department of Chemistry, Federal University of Piaui, Teresina, Brazil; 4 National Research Centre on Pomegranate, Solapur, Maharashtra, India; 5 Institute of Plant Genetics, Breeding and Biotechnology, University of Life Sciences in Lublin, Lublin, Poland

**Keywords:** 16SrVI group, brinjal little leaf disease, genetic diversity, PCR–RFLP, phylogenetic analysis, phytoplasma

## Abstract

Brinjal little leaf disease (BLL), caused by phytoplasmas, represents a major constraint to eggplant (*Solanum melongena* L.) production in India, leading to substantial yield losses. The present study aimed to assess the incidence of BLL in selected eggplant-growing regions of Maharashtra and Karnataka and to molecularly characterize the associated phytoplasma isolates using PCR–RFLP and phylogenetic analyses. Field surveys conducted across twelve locations during 2022–2023 revealed a high mean disease incidence of 43.12%, with considerable spatial variation ranging from 26.6% to 61.9%. Symptomatic plants exhibited typical features of phytoplasma infection, including reduced leaf size, chlorosis, virescence, witches’ broom, and severe stunting. Molecular detection using nested PCR consistently amplified phytoplasma-specific fragments confirming the presence of phytoplasmas in all analysed samples. PCR–RFLP analysis revealed limited genetic variability among most isolates, although distinct restriction profiles were observed for selected samples, particularly BLL-11. Cluster analysis grouped the isolates into two main clusters, indicating the presence of genetically differentiated lineages. Sequence analysis of the 16S rRNA gene and phylogenetic reconstruction demonstrated that all isolates belong to the 16SrVI ribosomal group and are closely related to “Candidatus *Phytoplasma trifolii*”. However, the formation of distinct phylogenetic clusters and haplotypes suggests the occurrence of previously uncharacterised genetic variants within this group. The median-joining haplotype network further supported recent diversification and local evolution of phytoplasma populations. These findings contribute to a better understanding of the genetic diversity and epidemiology of BLL-associated phytoplasmas and provide a foundation for future studies aimed at improving disease management strategies.

## 1 Introduction

Eggplant (*Solanum melongena* L.), a member of the Solanaceae family, is one of the most important vegetable crops cultivated in tropical and subtropical regions, particularly on the Indian subcontinent, where it is commonly referred to as brinjal (Alam et al., 2021). This crop has substantial economic and nutritional importance, as it constitutes a staple component of the diet in many countries and contributes significantly to farmers’ income (Bhalerao et al., 2025). India ranks second globally in eggplant production, accounting for approximately 21% of total world production, surpassed only by China (FAOSTAT, 2025). The high nutritional value of eggplant, attributed to its vitamin, mineral, and bioactive compound content, as well as its potential health-promoting properties, further highlights its importance in human nutrition (Bhaskar and Kumar, 2015).

Despite its considerable agricultural and nutritional significance, eggplant cultivation is severely constrained by various biotic factors, among which brinjal little leaf (BLL) disease is one of the most destructive. The disease was first described in India by (Thomas and Krishnaswami, 1939) and has since been widely reported in major eggplant-growing regions. BLL causes substantial yield losses, reaching up to 40% under moderate infection conditions, and may result in complete crop failure during severe epidemics (Mitra, 1993; Rao et al., 2018). Typical symptoms include reduced leaf size, virescence, phyllody, excessive branching (witches’ broom), and severe stunting, all of which markedly reduce the agronomic value of affected plants.

The etiological agents of BLL are phytoplasmas—specialised bacterial plant pathogens belonging to the class Mollicutes, characterised by the absence of a cell wall and an obligate parasitic lifestyle within the phloem tissues of plants and within the bodies of insect vectors (Doi et al., 1967; Bertaccini et al., 2014). Phytoplasmas possess highly reduced genomes and cannot be cultured on standard microbiological media, making their identification and characterisation largely dependent on molecular methods. Infection by phytoplasmas leads to a wide range of morphological and physiological abnormalities, including phyllody, virescence, witches’ broom, growth retardation, and sterility, primarily resulting from disruptions in plant hormonal regulation (Bertaccini, 2007).

Advances in molecular research have significantly improved our understanding of phytoplasma diversity and phylogenetic relationships, enabling the identification of multiple groups and subgroups associated with brinjal little leaf disease. Previous studies have demonstrated that BLL is associated with several phytoplasma groups, with the 16SrVI group—comprising, among others, “Candidatus *Phytoplasma trifolii*”—being the most frequently identified in India (Rao and Kumar, 2017). However, considerable genetic variability exists within this group, and the severity and nature of disease symptoms may vary depending on the phytoplasma strain, host species, plant developmental stage, and environmental conditions (McCoy, 1989; Bertaccini and Lee, 2018). Recent studies have demonstrated that phytoplasma-induced symptoms are mediated by secreted effector proteins, including SAP11 and SAP54, which interfere with plant hormonal signalling pathways and developmental regulators. SAP11 has been associated with witches’ broom formation through disruption of auxin and jasmonate signalling, whereas SAP54 promotes phyllody and floral abnormalities by targeting MADS-box transcription factors. These effectors are considered major determinants of symptom development and host manipulation in phytoplasma-infected plants (MacLean et al., 2011; Sugio et al., 2011). Despite numerous reports from different regions of India, knowledge of the genetic diversity of phytoplasmas associated with BLL in key eggplant-producing areas, such as Maharashtra and Karnataka, remains limited.

Therefore, the objective of the present study was to determine the incidence of brinjal little leaf disease in selected locations across Maharashtra and Karnataka, and to molecularly identify and characterise phytoplasma isolates using 16S rDNA markers, PCR-RFLP analysis, and phylogenetic methods. The findings are intended to expand current knowledge on the diversity of phytoplasmas causing BLL and to provide a foundation for further studies on disease epidemiology and the development of effective management strategies.

## 2 Materials and methods

### Survey of eggplant fields for disease incidence and sample collection

The incidence of Brinjal Little Leaf disease was determined based on morphological symptoms exhibited by diseased brinjal plants. The incidence of BLL disease was assessed by calculating the per cent incidence across all fields using the formula given below (Rathnamma and Patil, 2017).


$$\text{Disease Incidence }(\%)=\frac{\text{Number of symptomatic brinjal plants}}{\text{Total number of plants observed}}\times 100$$

**Figure 1:**
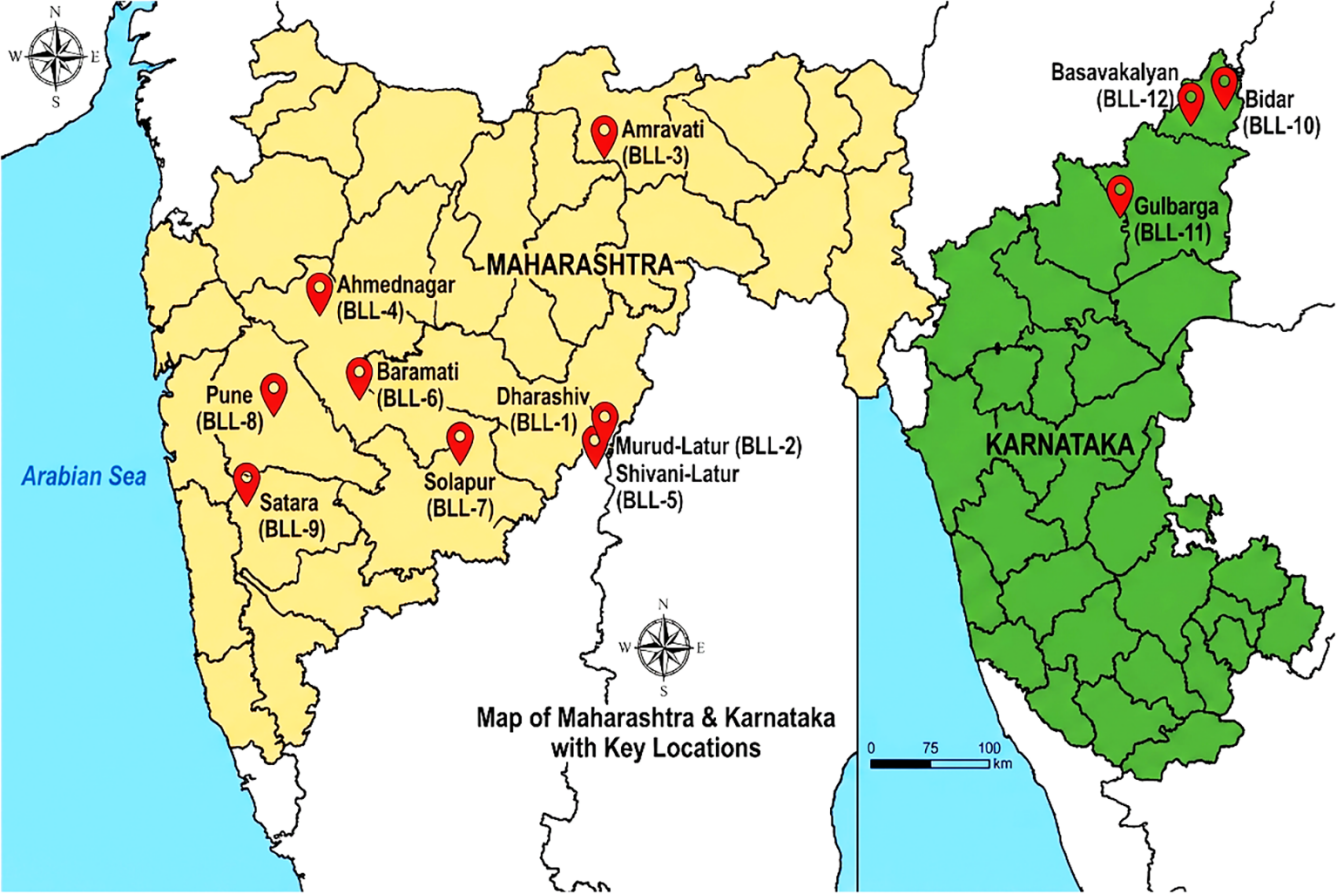
Survey sites of BLL sample collection.

Field surveys were conducted in 2022–2023 across 12 major brinjal-growing locations to assess disease incidence and collect symptomatic samples (**Figure 1**). At each location, three representative fields were selected, and at least 100 plants were assessed for disease symptoms.

**Figure 2:**
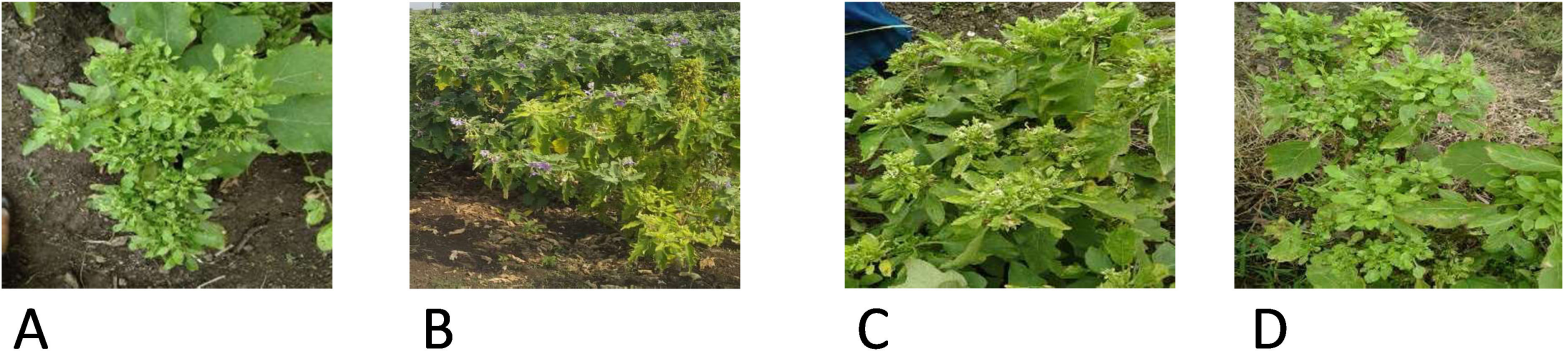
Symptoms of brinjal little leaf disease showing **(A)** Reduction in leaf size, **(B)** Chlorosis, **(C)** Reduction in leaf size, shortened petioles and internode, excessive branching, general stunting, **(D)** Witches’ broom on brinjal plants collected from different locations in Maharashtra and Karnataka states.

Symptomatic plant materials showing characteristic BLL symptoms, including reduction in leaf size, chlorosis, shortened petioles and internodes, excessive branching, general stunting, and Witches’ broom (**Figure 2**), were collected for molecular analysis. Fresh leaf samples were removed from symptomatic plants during sampling, immediately wrapped in sampling bags, and stored at -20 °C until DNA extraction was performed.

### PCR-based diagnosis of phytoplasma and PCR-RFLP analysis of 16S rDNA

Leaf samples from brinjal little leaf-infected plants were washed with 70% ethanol for 30 seconds, then with sterile distilled water for 1 minute, and dried on sterile tissue paper. Samples were chopped into small pieces and crushed in a mortar and pestle using liquid nitrogen. DNA was extracted using the CTAB buffer as described by Ahrens and Seemüller (1992). The first PCR was performed using phytoplasma-specific primers P1 (AAG AGT TTG ATC CTG GCT CAG GATT) and P7 (CGT CCT TCA TCG GCT CTT) (Smart et al., 1996). PCR Taq mixture (HiMedia) 2X (9 µL), Primer 10 pmol/µL (0.5 µL each) and template DNA 3 ng/μL (1 µL) with thermal profile: initial denaturation 95°C for 3 min 1 cycle, denaturation 95°C for 30 sec; annealing at 55°C for 30 sec; extension 72°C for 30 sec up to 30 cycles, final extension 72°C for 7 min and hold at 4°C for ∞. The amplified PCR product was diluted 1:80, and 1 µL of the DNA sample was used for nested PCR with primers R16F2n (GAA ACG ACT GCT AAG ACT GG) and R16R2 (TGA CGG GCG GTG TGT ACA AAC CCC G) (Gundersen and Lee, 1996), employing the same PCR conditions as before.

The nested PCR-amplified rDNA product from Phytoplasma isolates was subjected to restriction digestion with three restriction enzymes, *EcoRI*, *HaeIII*, and HindIII (Genei, Bangalore). EcoRI, HaeIII, and HindIII were selected based on previous reports demonstrating their ability to differentiate phytoplasma strains within the 16SrVI group and generate clear restriction profiles suitable for comparative analysis. HaeIII was included due to its high discriminatory power reported in earlier phytoplasma studies (Lee et al., 1998; Adkar-Purushothama et al., 2011).

Restriction digestion was performed in a mixture containing 10X Restriction assay buffer (1.5µl), 100X BSA (0.5µl), 2.5U Restriction enzymes (5U/µl), and Amplified PCR product (4µl). The reaction was carried out at 37°C for 8 hours in a PCR device (SensoQuest, Germany). The digested product was separated on a 4% agarose gel stained with ethidium bromide. 100bp and 1 kb ladders (Genei, Bangalore) were used as size markers. The data were entered into a binary matrix and analysed using Past software (Hammer et al., 2001). A dendrogram was constructed using the unweighted pair group method with arithmetic mean (UPGMA) cluster analysis algorithm, based on the Jaccard similarity coefficient.

### Nucleotide sequence-based analysis

The amplified 16S rDNA fragments of brinjal little leaf phytoplasma isolates were purified using a Qiagen column purification kit and sequenced by the Sanger method using an Applied Biosystems 3700 XL DNA Analyser (Eurofins Genomics India Private Limited, Bengaluru, India). The obtained nucleotide sequences were compared with reference from GenBank using the BLASTn and the iPhyClassifier online tool (https://plantpathology.ba.ars.usda.gov/cgi-bin/resource/iphyclassifier.cgi). All sequences were checked and, where necessary, manually edited using CLC Main Workbench 8.1.2 (QIAGEN, Aarhus, Denmark). For phylogenetic analysis, all sequences were aligned using the ClustalW algorithm with representative reference phytoplasma sequences retrieved from GenBank (**Supplementary Table 1**). Phylogenetic analyses of the concatenated and single-sequence data for maximum likelihood (ML) were performed using CLC Main Workbench 8.1.2 software (QIAGEN, Aarhus, Denmark) with the GTR+G model of nucleotide substitution and 1000 bootstrap replicates. *Acholeplasma laidlawii* was included as an outgroup to root the phylogenetic tree. NETWORK 10.2.0.0 (https://www.fluxus-engineering.com) was used to construct a median−joining (MJ) network (Bandelt et al., 1999) to describe relationships of the haplotypes.

## 3 Results

Infected plants exhibited typical symptoms of brinjal little leaf disease, including reduced leaf size, virescence, chlorosis, severe stunting, reduction of leaf lamina area, and the formation of witches’ broom. The mean disease incidence was 43.12% (**Table 1**). When disease incidence data were analysed by geographic origin of the samples, clear regional differences were observed. In Maharashtra, disease incidence showed wide spatial variation, ranging from 26.6% to 61.9% across different locations. In contrast, all surveyed locations in Karnataka exhibited moderate to high disease incidence, ranging from 40.47% to 53.3%, indicating a more uniform distribution of brinjal little leaf disease within this region.

**Table 1:** Morphological characteristics and percentage incidence of brinjal little leaf disease.

Sr. No.	Location	Total number of plants in the field	Infected plants	Incidence %	Stage of crop	Type of symptoms
Maharashtra						
1.	BLL-1	40	12	30.0	Post-harvest stage	Leaf reduction, phyllody, stunting
2.	BLL-2	30	8	26.6	Post-harvest stage	Leaf reduction, shortened internodes, bushy growth
3.	BLL-3	36	13	36.11	Flowering	Leaf reduction, stunting
4.	BLL-4	45	14	31.11	Fruit development	Leaf reduction
5.	BLL-5	50	28	56	Fruiting	Leaf reduction, phyllody, witches’ broom
6.	BLL-6	35	18	51.42	Fruit development	Leaf curling, bunchy growth, decline
7.	BLL-7	42	26	61.90	Flowering	Leaf reduction, chlorosis, stunting, phyllody
8.	BLL-8	43	21	48.8	Flowering	Leaf reduction, phyllody, bushy growth
9.	BLL-9	48	19	39.58	Fruit development	witch’s broom
KARNATAKA						
10.	BLL-10	38	16	42.10	Fruiting	Leaf reduction, stunting, phyllody
11.	BLL-11	15	8	53.3	Flowering	Leaf reduction, phyllody
12.	BLL-12	42	17	40.47	Flowering	Phyllody

Average Percentage of Disease Incidence = 43.12.

### Molecular detection of phytoplasmas

**Figure 3:**
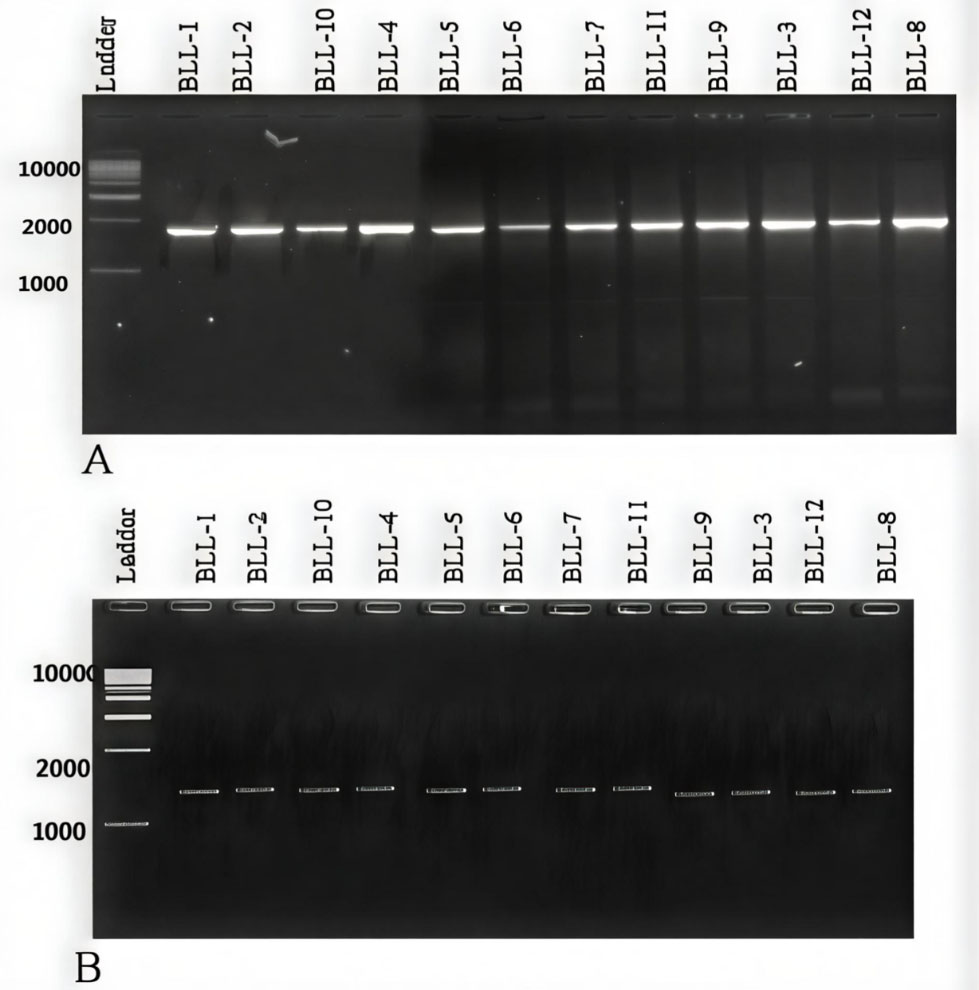
Agarose gel electrophoresis showing PCR products: **(A)** - from the first round (~1.8 kb) and **(B)** - nested PCR (~1.28 kb) for BLL-1–BLL-12 isolates.

Specific amplification of a ~1.8 kb fragment of the 16S rDNA gene was obtained in all eggplant samples exhibiting little leaf symptoms during the first round of PCR using the primer pair P1/P7. Subsequently, nested PCR performed with the primer pair R16F2n/R16R2 yielded an amplicon of approximately 1280 bp in all analysed samples (**Figure 3**). Negative controls consisting of DNA extracted from asymptomatic healthy brinjal plants and nuclease-free water (no-template control) were included in every PCR run to monitor for potential contamination.

### RFLP analysis and grouping of phytoplasma isolates

Nested PCR products of approximately 1280 bp were subjected to restriction digestion with the enzymes *HindIII*, *EcoRI*, and *HaeIII*. The restriction profiles obtained with *HindIII* and *EcoRI* were largely uniform among most isolates (**Table 2**). The *HindIII* enzyme did not digest the amplicon in eleven isolates, whereas isolate BLL-11 produced two fragments of 800 bp and 480 bp. Following digestion with *EcoRI*, eleven isolates exhibited two fragments of 750 bp and 530 bp, while isolate BLL-8 lacked a restriction site for this enzyme and remained undigested (1280 bp). The greatest variation in restriction profiles was observed after digestion with *HaeIII*. Eleven isolates yielded two fragments of 1090 bp and 190 bp, whereas isolate BLL-11 displayed a distinct profile consisting of three fragments of 750 bp, 340 bp, and 190 bp.

**Table 2:** Restriction fragment size (in base pairs) of brinjal little leaf sample.

Sr. no.	Location	Enzyme		
		*Hind* III	*EcoRI*	*Hae* III
1.	BLL-1	1280	750, 530	1090, 190
2.	BLL-2	1280	750, 530	1090, 190
3.	BLL-3	1280	750, 530	1090, 190
4.	BLL-4	1280	750, 530	1090, 190
5.	BLL-5	1280	750, 530	1090, 190
6.	BLL-6	1280	750, 530	1090, 190
7.	BLL-7	1280	750, 530	1090, 190
8.	BLL-8	1280	1280	1090, 190
9.	BLL-9	1280	750, 530	1090, 190
10.	BLL-10	1280	750, 530	1090, 190
11.	BLL-11	480, 800	750, 530	750, 340, 190
12.	BLL-12	1280	750, 530	1090, 190

**Figure 4:**
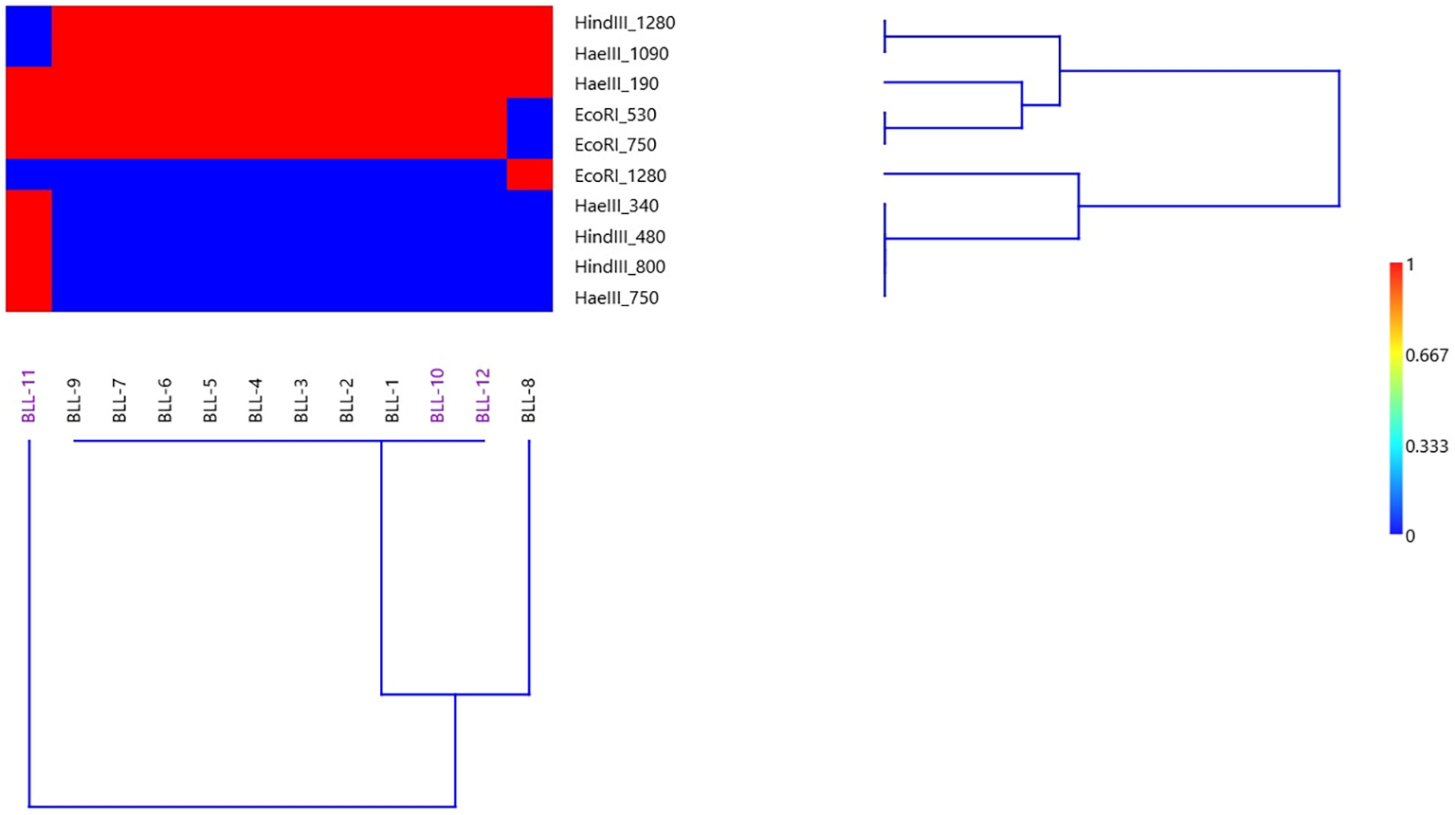
UPGMA dendrogram showing the genetic relationships among phytoplasma isolates associated with brinjal little leaf disease based on RFLP analysis.

Cluster analysis based on Jaccard’s similarity coefficients, performed using the UPGMA method, separated the 12 phytoplasma isolates into two major clusters at a similarity level of 33.4% (**Figure 4**). Cluster I comprised the majority of isolates, including BLL-1, BLL-2, BLL-3, BLL-4, BLL-5, BLL-6, BLL-7, BLL-9, BLL-10, and BLL-12, which exhibited high genetic similarity, with similarity coefficients exceeding 0.95, indicating nearly identical restriction profiles. Within this cluster, isolate BLL-8 formed a distinct subcluster, joining the main group at a similarity level of approximately 0.52, reflecting moderate genetic divergence compared to the other isolates.

Cluster II consisted exclusively of isolate BLL-11, which showed the lowest similarity to all other isolates and clustered separately at a similarity level of approximately 0.33. This distinct position corresponds to its unique restriction profile observed in the RFLP analysis. Overall, the dendrogram revealed low genetic variability among most isolates, except for BLL-8 and, particularly, BLL-11, which showed greater genetic divergence and may represent distinct phytoplasma variants.

### Sequencing and sequence analysis of the 16S rDNA gene

All amplicons were subjected to sequencing, of which ten (BLL-1, BLL-2, BLL-3, BLL-4, BLL-5, BLL-7, BLL-8, BLL-9, BLL-10, and BLL-12) were characterised by satisfactory sequence quality and were therefore used for further analyses. The obtained 16S rDNA gene sequences were deposited in the GenBank database under accession numbers PP869765, PP861156, PP910331, PP861313, PP862645, PP864142, PP869766, PP869767, PP869768 and PP869769.

BLASTn analysis demonstrated that the isolates’ sequences exhibited high similarity to phytoplasma sequences deposited in GenBank, with nucleotide identities ranging from 94.81% to 99.83% (E = 0.0). Analysis performed using the iPhyClassifier tool indicated that the sequences of the studied isolates showed 98.04% identity with the reference sequence of “Candidatus *Phytoplasma trifolii*” (AY390261), indicating their affiliation with the 16SrVI ribosomal group.

Phylogenetic analysis was performed using 16S rDNA gene sequences, together with reference sequences representing different phytoplasma groups and subgroups deposited in GenBank. At the same time, *Acholeplasma laidlawii* (U14905) was used as an outgroup to root the tree.

**Figure 5:**
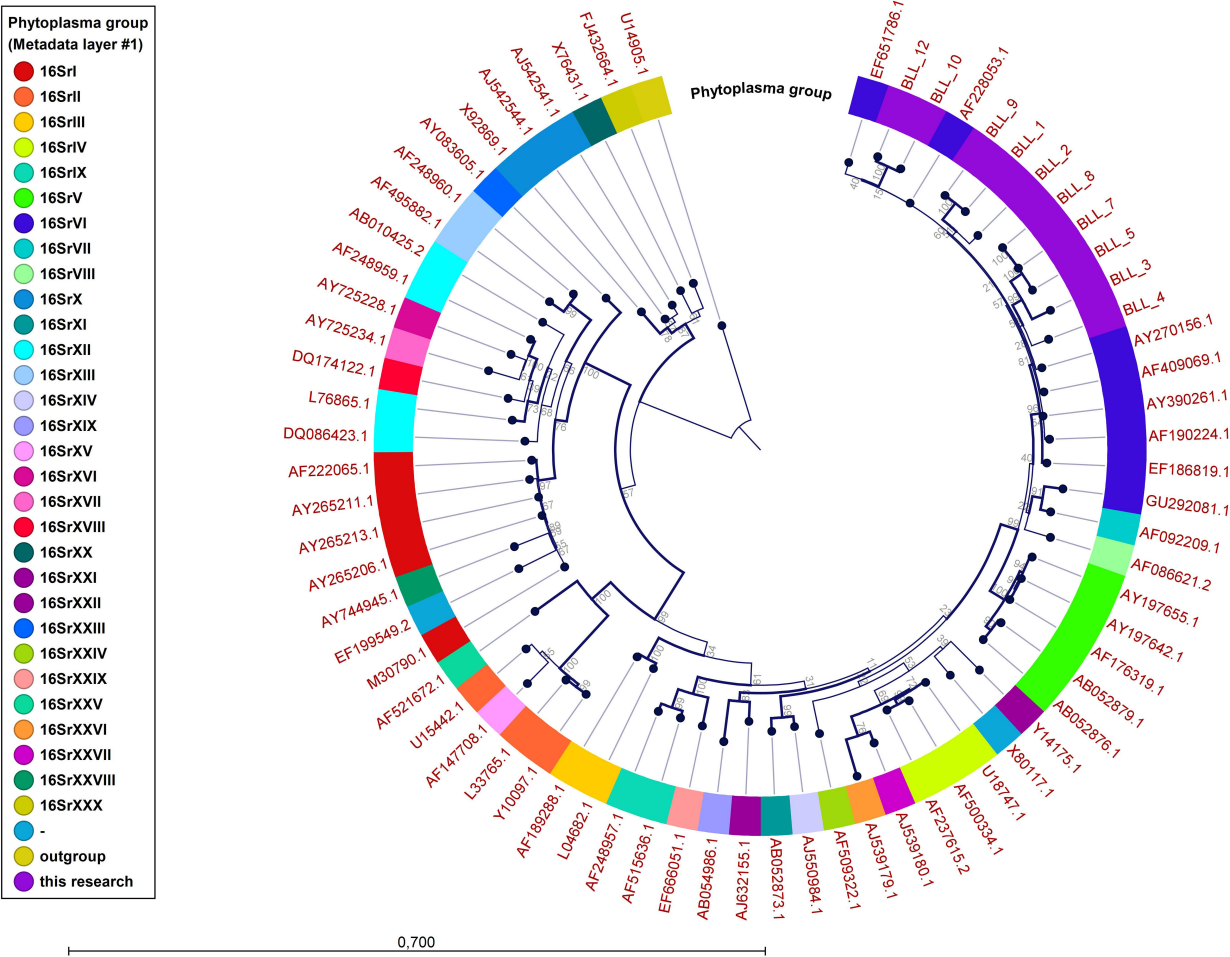
Phylogenetic tree based on 16S rRNA gene sequences showing the relationships between brinjal small leaf phytoplasma isolates and reference sequences, constructed using the maximum likelihood (ML) method.

The obtained phylogenetic tree (**Figure 5**) showed that all analysed phytoplasma isolates associated with eggplant little leaf grouped within the clade corresponding to the 16SrVI (Clover proliferation group). This clade was clearly separated from the remaining phytoplasma groups and supported by a high bootstrap value of 96%, confirming the stability and reliability of the obtained tree topology.

**Figure 6:**
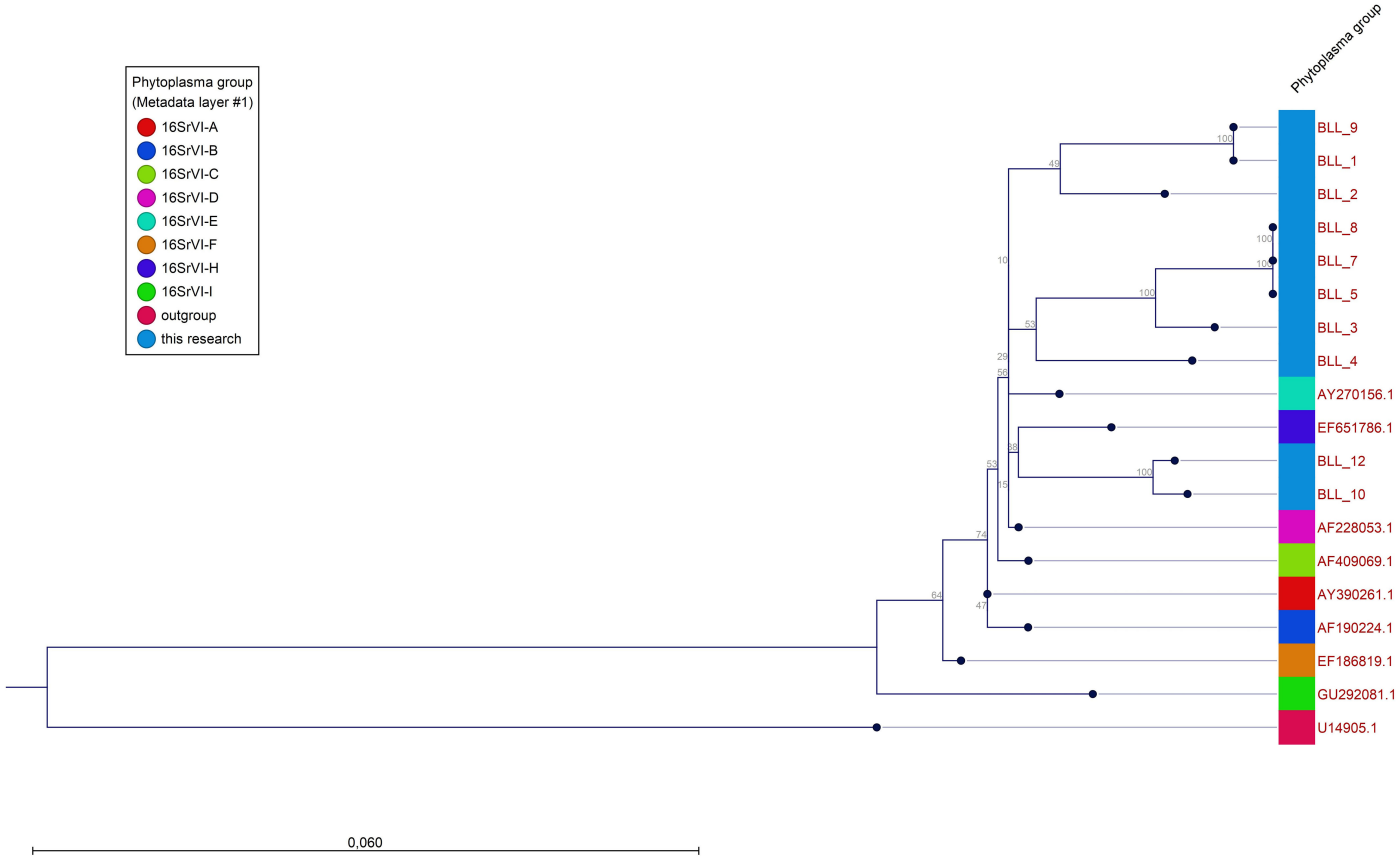
Phylogenetic tree based on 16S rRNA gene sequences showing the relationships between brinjal small leaf phytoplasma isolates and reference sequences 16Sr_VI group, constructed using the maximum likelihood (ML) method.

Within the 16SrVI clade, clear internal differentiation among the studied isolates was observed (**Figure 6**). Isolates BLL-10 and BLL-12 were grouped near the reference sequence representing subgroup 16SrVI-H, indicating their affiliation with previously described phylogenetic lineages. At the same time, isolates BLL-2, BLL-1, and BLL-9 formed a coherent internal phylogenetic cluster, characterised by high mutual sequence similarity (>97.5%) and clear separation from the known reference subgroups of the 16SrVI group. Similarly, isolates BLL-4, BLL-3, BLL-5, BLL-7, and BLL-8 formed a separate subgroup within the 16SrVI cluster. This pattern suggests the presence of several distinct genetic lineages of eggplant little leaf phytoplasmas in the studied regions.

The topology of the phylogenetic tree was consistent with sequence-similarity results obtained using BLASTn and with classification performed using the iPhyClassifier tool, confirming the affiliation of the analysed isolates with the 16SrVI group and indicating the presence of divergent haplotypes or new genetic variants within this ribosomal group.

**Figure 7:**
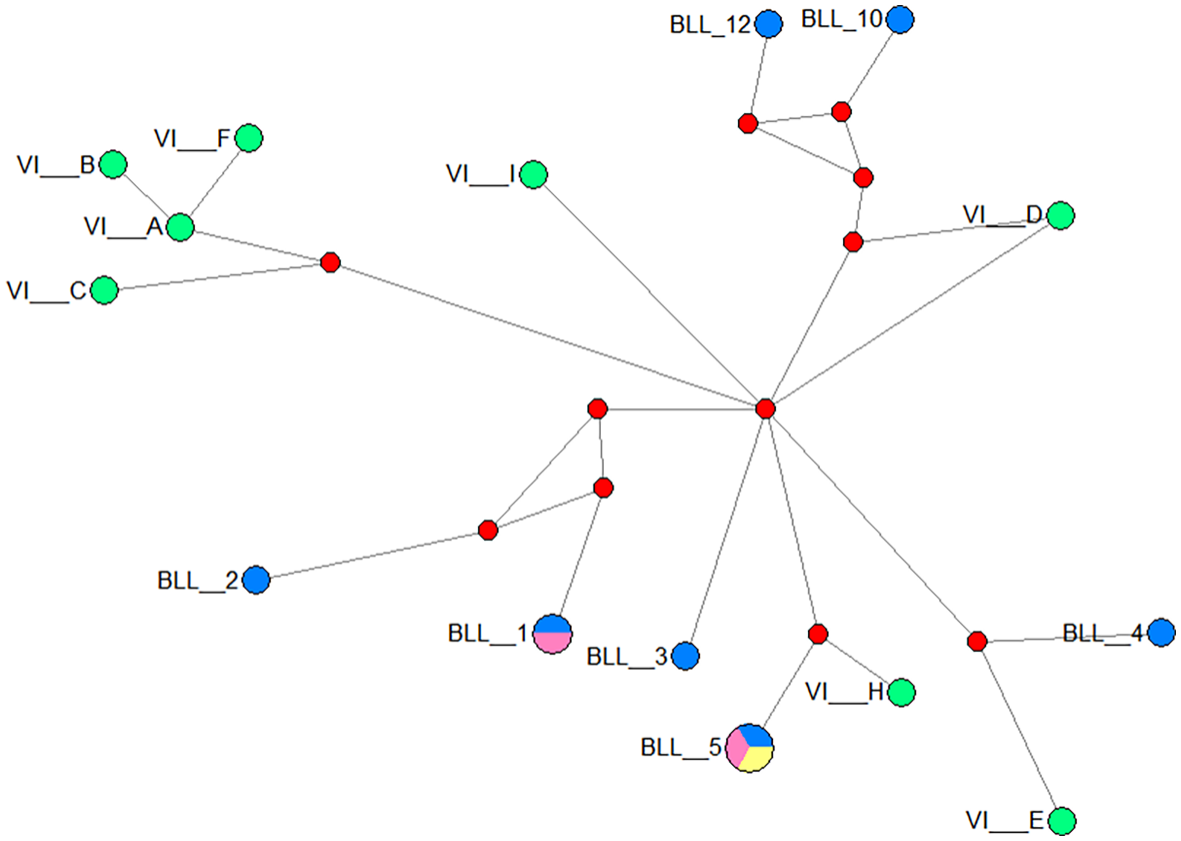
Median-joining haplotype network based on 16S rDNA sequences of phytoplasma isolates associated with eggplant little leaf disease and reference sequences. Yellow/rose/blue circles = sampled isolates; green reference subgroup haplotypes; red circles labelled = median vectors (inferred intermediate haplotypes). Circle area is proportional to haplotype frequency. Lines denote single mutational steps (short lines) or multiple steps (longer series of connections). The central node represents the inferred ancestral/consensus haplotype from which several derived haplotypes radiate.

A median-joining haplotype network was constructed from the aligned 16S rDNA sequences to visualise fine-scale genetic relationships among the studied phytoplasma isolates and reference sequences (**Figure 7**). The network shows a central median node (mv2) from which multiple branches radiate, indicating a star-like topology. A cluster of reference haplotypes corresponding to subgroups VI_A/VI_B/VI_C/VI_F is separated from the central node by one intermediate median vector, indicating close but non-identical relationships to the sampled isolates. Reference haplotypes representing subgroups VI_D and VI_E are connected to separate peripheral branches, each linked to the central node through short mutational steps. Isolate BLL_4 is associated closely with the VI_E branch. Two isolates (BLL_10 and BLL_12) form a distinct, tightly clustered group at the top-right of the network, separated from reference sequences by multiple median vectors, consistent with a distinct haplotype lineage.

A set of isolates (BLL_1and BLL_9, BLL_2, BLL_3, as well as BLL_5, BLL_7 and BLL_8) occupy separate but neighbouring branches around the central node; pie charts in several nodes indicate that some haplotypes are shared among samples from multiple sampling locations (mixed colours), suggesting haplotype sharing or recent dispersal among regions. Numerous median vectors are present between sampled haplotypes, representing inferred unsampled or ancestral haplotypes and implying that the sampled sequences capture only part of the population’s diversity.

## 4 Discussion

The conducted studies demonstrated a high incidence of brinjal little leaf disease (BLL) in the investigated regions of India, ranging from 26.6% to 61.9%. These findings confirm that the disease poses a significant threat to eggplant production in Maharashtra and Karnataka, which are among the main regions of cultivation. A similar range of variation in disease incidence was reported by Rathnamma and Patil (2017), who documented BLLs ranging from 2% to 95% by location. Likewise, Venkataravanappa et al. (2022) indicated the widespread distribution of the disease in different regions of India. The relatively high disease incidence observed in intensively cultivated agricultural areas may indicate favourable conditions for the persistence and spread of phytoplasmas. Factors such as continuous cropping, the presence of alternative host plants acting as reservoirs of the pathogen, and the occurrence of insect vectors may play an important role in the epidemiology of the disease, as confirmed by previous studies (Bertaccini and Lee, 2018; Rao et al., 2018).

The characteristic symptoms observed in infected plants, including reduced leaf size, chlorosis, phyllody, witches’ broom, and severe stunting, are consistent with those reported for phytoplasma infections in eggplant and other host species (Bertaccini et al., 2014; Rao and Kumar, 2017). These developmental abnormalities are closely linked to disturbances in phytohormone-regulated pathways. Recent studies have highlighted the central role of auxin-mediated signalling in coordinating plant growth, organ development, and responses to biotic stress (Ali et al., 2025). Phytoplasmas are known to manipulate host developmental pathways through effector proteins that interfere with hormone homeostasis and transcriptional regulation, resulting in symptoms such as excessive shoot proliferation, leaf deformation, and floral abnormalities. Disruption of auxin signalling, together with interactions involving jasmonic acid and salicylic acid pathways, may contribute substantially to symptom expression and disease susceptibility (Ali et al., 2025). Understanding the molecular mechanisms underlying these interactions may provide valuable targets for future breeding and disease-management strategies.

Molecular detection using nested PCR enabled the effective identification of phytoplasmas in all analysed samples exhibiting disease symptoms. The amplification of products of the expected size (~1.8 kb in the first PCR round and ~1.28 kb in the nested reaction) confirms the high sensitivity and usefulness of this method in phytoplasma diagnostics. These results are consistent with previous studies in which nested PCR was successfully used to detect phytoplasmas in eggplant and other cultivated plants (Abirami et al., 2022; Gawande et al., 2022). The high sensitivity of this method results from its ability to detect small amounts of pathogen DNA in phloem tissues, which is particularly important for phytoplasmas that cannot be cultured in the laboratory (Bertaccini et al., 2014).

PCR–RFLP analysis revealed genetic diversity among the studied phytoplasma isolates. Among the restriction enzymes used, HaeIII showed the highest ability to differentiate restriction profiles, indicating its particular usefulness in the analysis of phytoplasma genetic variability. Similar results were obtained by Lee et al. (1998) and confirmed by subsequent studies Adkar-Purushothama et al. (2011); Şimşek and Güldür (2021); Mutaqin et al. (2023), demonstrating the high effectiveness of PCR–RFLP analysis in phytoplasma classification. The clustering of isolates obtained in the UPGMA analysis indicated the presence of distinct genetic lineages. Importantly, the clustering pattern obtained from the RFLP analysis was consistent with the results of the phylogenetic analysis based on 16S rDNA gene sequences, further confirming the reliability of the results and indicating the actual genetic diversity of phytoplasma populations associated with BLL disease.

Sequence analysis of the 16S rDNA gene and reconstruction of the phylogenetic tree demonstrated that all analysed isolates belong to the 16SrVI ribosomal group and are closely related to the species “Candidatus *Phytoplasma trifolii*”. This finding is consistent with earlier reports indicating that phytoplasmas belonging to the 16SrVI group constitute the dominant etiological agent of eggplant little leaf disease in various regions of India (Yadav et al., 2015; Kumar et al., 2017; Dutta et al., 2022; Karthikeyan et al., 2024).

Despite the clear affiliation of all studied isolates with the 16SrVI group, detailed phylogenetic analysis revealed significant genetic diversity within this group. Several isolates formed well-supported clusters that were clearly separated from previously described reference subgroups, indicating the presence of genetically differentiated lineages within BLL-associated phytoplasma populations. These findings highlight the complex population structure of phytoplasmas infecting eggplant in India and suggest ongoing diversification within the 16SrVI group.

According to current phytoplasma classification criteria, the establishment of a new 16Sr subgroup requires virtual RFLP analysis of the F2n/R2 region of the 16S rRNA gene and a similarity coefficient below 0.97 relative to previously recognised subgroups (Lee et al., 1998; Wei and Zhao, 2022). However, because closely related phytoplasma strains often exhibit limited divergence within the 16S rRNA locus, multilocus sequence analysis (MLSA) based on additional loci such as secA, tuf, rp, and groEL is considered essential for robust taxonomic classification and improved phylogenetic resolution (Wei and Zhao, 2022). Therefore, the present results should be interpreted as evidence of genetic differentiation rather than formal subgroup designation. Nevertheless, the identification of distinct phylogenetic clusters contributes valuable information regarding the diversity and population structure of phytoplasmas associated with BLL disease.

Additional insight into the genetic relationships among the analysed isolates was provided by the median-joining haplotype network constructed from the 16S rDNA sequences. The star-like topology observed in the network is characteristic of populations that have undergone recent expansion from a common ancestral haplotype, followed by local diversification through mutation accumulation (Bandelt et al., 1999). The distribution of isolates across several branches radiating from the central haplotype indicates the coexistance of multiple closely related lineages within the phytoplasma population infecting eggplant in the investigated regions. Importantly, the isolates clustered near Several isolates were separated from reference haplotypes by intermediate median vectors, suggesting the existence of unsampled ancestral forms or additional genetic variants. Such patterns may result from founder effects, local evolutionary processes, and vector-mediated dispersal among geographically connected cultivation areas. Similar population structures have been reported for other phytoplasma-associated diseases and are frequently associated with a combination of host adaptation, vector movement, and regional ecological factors (Bertaccini and Lee, 2018; Rao et al., 2018). Furthermore, the clustering of several isolates near different reference subgroups of the 16SrVI group supports the view that phytoplasma populations associated with BLL are dynamic and continuously evolving under natural field conditions.

The considerable geographic variation in disease incidence observed in the present study (26.6–61.9%) likely reflects the combined influence of environmental and agronomic factors. Continuous cropping systems, the presence of alternative weed hosts, climatic conditions favouring vector survival, and differences in crop management practices may all contribute to regional variation in disease pressure (Bertaccini and Lee, 2018; Rao et al., 2018). Karnataka exhibited a slightly higher average disease incidence than Maharashtra, which may be associated with climatic conditions favourable for leafhopper populations and the intensive cultivation of vegetable crops. However, detailed epidemiological studies integrating vector abundance, environmental variables, and cropping practices are required to verify these relationships.

Although vector surveys were not conducted in the present study, previous investigations in India have identified *Hishimonus phycitis* (Distant) and *Amrasca biguttula biguttula* (Ishida) as important leafhopper vectors associated with the transmission of 16SrVI phytoplasmas in brinjal (Kumar et al., 2017). Differences in vector abundance, seasonal activity, and movement among agricultural landscapes may therefore contribute to the geographic variation in disease incidence observed in the surveyed regions. Further studies integrating phytoplasma detection with entomological monitoring will be essential for understanding disease epidemiology and transmission dynamics.

Several limitations of the present study should be acknowledged. First, only ten of the twelve collected isolates yielded sequences of sufficient quality for downstream analyses, which may not fully capture the genetic diversity of phytoplasma populations present in the surveyed regions. Second, taxonomic interpretation was based exclusively on the 16S rRNA gene, whose discriminatory power among closely related phytoplasma lineages is limited (Lee et al., 1998; Wei and Zhao, 2022). Third, no surveys of insect vector populations were conducted; therefore, the influence of vector abundance and distribution on regional disease incidence could not be assessed. Finally, the present study did not evaluate potential differences in pathogenicity, symptom severity, host range, or transmission efficiency among the identified haplotypes. Controlled transmission experiments using insect vectors or dodder would be required to determine whether the observed genetic differentiation corresponds to biologically meaningful differences in virulence or host specificity.

Future studies should prioritise multilocus sequence analysis (MLSA) using protein-coding genes such as secA, tuf, rp, and groEL to validate the phylogenetic distinctness of the divergent lineages identified here and improve taxonomic resolution. Whole-genome sequencing of representative isolates, particularly the genetically divergent BLL-11 isolate and the BLL-10/BLL-12 lineage, would further clarify evolutionary relationships and facilitate the identification of virulence-associated genes and phytoplasma effectors. Such genomic resources would also support the development of highly specific molecular diagnostic markers and genome-informed disease-management strategies.

Looking ahead, emerging technologies such as single-cell RNA sequencing (scRNA-seq) and spatial transcriptomics offer exciting opportunities to investigate phytoplasma–host interactions at unprecedented resolution (Hu et al., 2025; Ali et al., 2026). These approaches can reveal cell-type-specific responses within infected phloem tissues, identify molecular pathways involved in symptom development, and improve our understanding of hormone-mediated regulatory networks disrupted by phytoplasma infection. For BLL-affected eggplant, the integration of genomics, transcriptomics, vector ecology, and spatial omics approaches could substantially advance our understanding of phytoplasma biology and provide new opportunities for sustainable disease control.

## 5 Conclusions

The study confirmed a high incidence of brinjal little leaf disease (BLL) in selected regions of Maharashtra and Karnataka, with an average disease incidence exceeding 43%. These results indicate that BLL remains an important factor limiting eggplant production in southern and western India.

Molecular analyses using nested PCR, PCR-RFLP, and sequencing of the 16S rRNA gene unequivocally confirmed the presence of phytoplasmas belonging to the 16SrVI ribosomal group, closely related to “Candidatus *Phytoplasma trifolii*”. At the same time, the results revealed clear genetic diversity among the studied isolates, both in RFLP analysis and in phylogenetic reconstruction.

The median-joining haplotype network provided additional insight into the relationships among the analysed isolates and supported the presence of multiple closely related haplotypes within the phytoplasma population associated with eggplant little leaf disease. The star-like structure of the network and the occurrence of several distinct haplotype clusters indicate ongoing diversification of phytoplasma populations within the 16SrVI group. These findings further confirm the complex genetic structure of the pathogen population in the investigated regions.

The observed separation of some isolates within the 16SrVI clade, together with their distinct position in the haplotype network, may indicate ongoing diversification and regional evolution within 16SrVI phytoplasma populations associated with brinjal little leaf disease in India. However, the final confirmation of their taxonomic status requires expanded multilocus sequence analysis (MLSA) and broader geographical sampling.

Overall, the obtained data contribute to a better understanding of the genetic diversity and population structure of phytoplasmas associated with eggplant little leaf disease in India. The results also provide a basis for further research on disease epidemiology, the role of insect vectors, and the development of effective management strategies. Future studies integrating multilocus molecular analyses, vector population studies, and broader geographic sampling will be essential for a comprehensive understanding of the mechanisms driving the spread and diversification of BLL-associated phytoplasmas under field conditions.

## Data Availability

The original contributions presented in the study are included in the article/**Supplementary Material**, further inquiries can be directed to the corresponding author/s.
