# Supplementary material for: Incidence, molecular characterization and phylogenetic diversity of phytoplasmas associated with brinjal little leaf disease in Maharashtra and Karnataka, India
Source: Front Plant Sci. 2026 Jun 29;17:1870998. doi: 10.3389/fpls.2026.1870998 (PMC13366124; doi:10.3389/fpls.2026.1870998)
Supplement: Supplementary file 1 [file Table1.docx]

**Supplementary Table 1: Phytoplasma 16S rDNA gene sequences used for phylogenetic analysis.**

| **Sr.No.** | **Strain Name** | **PhytoplasmaGroup** | **GenBank accession number** | **Reference** |
| --- | --- | --- | --- | --- |
| 1. | Oenothera phytoplasma 86-7 | 16SrI-B | M30790 | Lee et al., 2004a |
| 2. | Clover PhyllodyPhytoplasma | 16SrI-C | AF222065 | Lee et al., 1998 |
| 3. | Aster yellows phytoplasma | 16SrI-D | AY265206 | Lee et al., 1998 |
| 4. | Blue berry stunt phytoplasma | 16SrI-E | AY265213 | Lee et al., 1998 |
| 5. | **Aster yellows phytoplasma** | 16SrI-F | AY265211 | Lee et al., 1998 |
| 6. | Peanut witches’-broom phytoplasma | 16SrII-A | L33765 | Lee et al., 1998 |
| 7. | Ca.Phytoplasma lycopersici | - | EF199549 | Arocha al., 2007 |
| 8. | Candidatus Phytoplasma citri | 16SrII-B | U15442 | Zreik et al., 1995 |
| 9. | Candidatus Phytoplasma australasia | 16SrII-D | Y10097 | White et al., 1998 |
| 10. | Western X phytoplasma | 16SrIII-A | L04682 | Schneider et al.,1995 |
| 11. | Clover yellow edge phytoplasma | 16SrIII-B | AF189288 | Lee et al.,1998 |
| 12. | Coconut lethal yellowing phytoplasma | 16SrIV-A | U18747 | Harrison et al., 1994 |
| 13. | Phytoplasma sp. LfY5(PE65)-Oaxaca | 16SrIV-B | AF500334 | Harrison et al., 2002; Wei et al., 2007 |
| 14. | Carludovica palmata leaf yellowing phytoplasma | 16SrIV-D | AF237615 | Harrison et al.,2002; Wei et al., 2007 |
| 15. | ‘Ca. Phytoplasma ulmi’ | 16SrV-A | AY197655 | Lee et al., 2004b |
| 16. | ‘Ca. Phytoplasma ziziphi’ JWB-G1 | 16SrV-B | AB052876 | Jung et al., 2003a |
| 17. | Ca. Phytoplasma vitis’ | 16SrV-C | AF176319 | Davis and Dally, 1999 |
| 18. | Alder yellows phytoplasma | 16SrV-C | AY197642 | Lee et al. , 1998 |
| 19. | Ca. Phytoplasma ziziphi’ | 16SrV-G | AB052879 | Jung et al. , 2003a; Wei et al., 2007 |
| 20. | Ca. Phytoplasma trifolii’ | 16SrVI-A | AY390261 | Hiruki& Wang , 2004 |
| 21. | 'Fragaria multicipita' phytoplasma | 16SrVI-B | AF190224 | Jomantiene, et al.,1998 |
| 22. | Clover proliferation phytoplasma | 16SrVI-C | AF409069 | Jacob et al., 2003 |
| 23. | Periwinkle little leaf phytoplasma | 16SrVI-D | AF228053 | Siddique et al., 2001 |
| 24. | Centaurea solstitialis virescence phytoplasma | 16SrVI-E | AY270156 | Faggioli et al., 2004 |
| 25. | Catharanthus phyllody phytoplasma | 16SrVI-F | EF186819 | Martini et al., 2007 |
| 26. | Portulaca little leaf phytoplasma | 16SrVI-H | EF651786 | Samad et al., 2008 |
| 27. | Candidatus Phytoplasma australamericanum | 16SrVI-I | GU292081 | Valiunas et al., 2009 |
| 28. | Candidatus Phytoplasma fraxini | 16SrVII-A | AF092209 | Griffiths et al., 1999 |
| 29. | Candidatus Phytoplasma luffae | 16SrVIII-A | AF086621 | Griffiths et al., 1999 |
| 30. | Candidatus Phytoplasma phoenicium | 16SrIX-D | AF515636 | Verdin et al., 2002 |
| 31. | Pigeon pea witches'-broom phytoplasma | 16SrIX-A | AF248957 | Davis and Dally, 2000 |
| 32. | Candidatus Phytoplasma mali | 16SrX-A | AJ542541 | Seemu¨ller and Schneider, 2004 |
| 33. | Phytoplasma sp. | 16SrX-D | X92869 | Marcone et al. , 2004a |
| 34. | Candidatus Phytoplasma prunorum | 16SrX-F | AJ542544 | Seemu¨ller and Schneider, 2004 |
| 35. | Candidatus Phytoplasma oryzae | 16SrXI-A | AB052873 | Jung et al., 2003b |
| 36. | Candidatus Phytoplasma solani | 16SrXII-A | AF248959 | Davis and Dally, 2000 |
| 37. | 'Allocasuarina muelleriana' phytoplasma | - | AY135523 | Gibb et al., 1995 |
| 38. | Candidatus Phytoplasma australiense | 16SrXII-B | L76865 | Davis et al., 1997 |
| 39. | Candidatus Phytoplasma japonicum | 16SrXII-D | AB010425 | Sawayanagi et al., 1999 |
| 40. | Candidatus Phytoplasma fragariae | 16SrXII-E | DQ086423 | Valiunas et al.,2006 |
| 41. | Mexican periwinkle virescence phytoplasma | 16SrXIII-A | AF248960 | Harrison et al., 2002 |
| 42. | Chinaberry yellows phytoplasma | 16SrXIII | AF495882 | Harrison et al., 2002 |
| 43. | Bermuda grass white leaf phytoplasma | 16SrXIV-A | AJ550984 | Marcone et al., 2004b |
| 44. | Candidatus Phytoplasma brasiliense | 16SrXV-A | AF147708 | Montano et al., 2001 |
| 45. | Candidatus Phytoplasma graminis | 16SrXVI-A | AY725228 | Arocha et al., 2005 |
| 46. | Candidatus Phytoplasma caricae | 16SrXVII-A | AY725234 | Arocha et al., 2005 |
| 47. | Candidatus Phytoplasma americanum | 16SrXVIII-A | DQ174122 | Lee et al., 2006 |
| 48. | Candidatus Phytoplasma castaneae | 16SrXIX-A | AB054986 | Jung et al.,2002; Wei et al.,2007 |
| 49. | Candidatus Phytoplasma rhamni | 16SrXX-A | X76431 | Marcone et al., 2004a; Wei et al., 2007 |
| 50. | Candidatus Phytoplasma pini | 16SrXXI-A | AJ632155 | Schneider et al., 2005; Wei et al., 2007 |
| 51. | Awka wilt phytoplasma | 16SrXXII-A | Y14175 | Wei et al., 2007 |
| 52. | Phytoplasma sp. | - | X80117 | Tymon et al., 1998 |
| 53. | Buckland Valley grapevine yellows phytoplasma | 16SrXXIII-A | AY083605 | Wei et al., 2007 |
| 54. | Sorghum bunchy shoot phytoplasma | 16SrXXIV-A | AF509322 | Wei et al., 2007 |
| 55. | Weeping tea tree witches'-broom phytoplasma | 16SrXXV-A | AF521672 | Wei et al., 2007 |
| 56. | Sugarcane phytoplasma | 16SrXXVI-A | AJ539179 | Wei et al., 2007 |
| 57. | Sugarcane phytoplasma | 16SrXXVII-A | AJ539180 | Wei et al., 2007 |
| 58. | Derbid phytoplasm | 16SrXXVIII-A | AY744945 | Wei et al., 2007 |
| 59. | Candidatus Phytoplasma omanense | 16SrXXIX-A | EF666051 | Al-Saady et al.,2008 |
| 60. | Salt cedar witches'-broom phytoplasma | 16SrXXX | FJ432664 | Zhao et al.,2009 |
| 61. | *Acholeplasma ladwii* | - | U14905.1 | Artiushin et al., 1995; Zhao et al.,2009 |
